# An Eye Tracking Based Framework for Safety Improvement of Offshore Operations

**DOI:** 10.16910/jemr.16.3.2

**Published:** 2023-08-10

**Authors:** Muhammad A. Raza, Raj Kiran, Saima Ghazal, Ziho Kang, Saeed Salehi, Edward Cokely, Jiwon Jeon

**Affiliations:** University of Engineering & Technology, Lahore, Pakistan; School of Petroleum and Geological Eng., University of Oklahoma, USA; Inst. of Applied Psychology, University of the Punjab, Pakistan; Department of Industrial & Systems Eng., University of Oklahoma, USA; Department of Psychology, University of Oklahoma, USA

**Keywords:** Eye tracking, gaze, situational awareness, saccades, individual differences, safety, offshore operations

## Abstract

Offshore drilling operations consist of complex and high-risk processes. Lack of situational awareness in drilling operations has become an important human factor issue that causes safety accidents. Prolonged work shifts and fatigue are some of the crucial issues that impact performance. Eye tracking technology can be used to distinguish the degree of awareness or alertness of participants that might be related to fatigue or onsite distractions. Oculomotor activity can be used to obtain visual cues that can quantify the drilling operators’ situational awareness that might enable us to develop warning alarms to alert the driller. Such systems can help reduce accidents and save non-productive time. In this paper, eye movement char-acteristics were investigated to differentiate the situational awareness between a representa-tive expert and a group of novices using a scenario-based Virtual Reality Drilling Simulator. Significant visual oculomotor activity differences were identified between the expert and the novices that indicate an eye-tracking based system can detect the distraction and alert-ness exhibited by the workers. Results show promise on developing a framework which implements a real-time eye tracking technology in various drilling operations at drilling rigs and Real Time Operation Centers to improve process safety.

## Introduction

Offshore energy extraction processes are overly complex systems that involve risky operations. Real-time data monitoring is a unique and highly discussed concept which has caught the imagination of every industry and academia in recent days. Dynamic data monitoring and feedback systems have taken a leap with the technological advancements in cloud computing and data mining techniques. In a typical application, the real-time system receives the onsite data from sensors, stores it in a database, processes the data using sophisticated algorithms, and finally provides the output in the form of alerts or feedback to the users. These feedback systems are now being used in many industries such as meteorology, hydrology, and the health sector. The real-time monitoring systems in the petroleum industry have also been consistently on the upsurge. The concept was laid back in the 1980s, but due to lack of technological capabilities and wide acceptance, it was not at the focal point of industrial operation. The breakthroughs in real-time data technology have allowed the improvement in the quality of remote monitoring infrastructure and now several remote centers such as Drilling Command and Control System, Drilling Data Center, Real Time Operations Center (RTOC) have been established to improve the safety and efficiency of the onsite operations (6; 21; 58). In the early days, the focus was to monitor the operation and simultaneously provide expert assistance to onsite personnel. However, with time the application has spread further and included informed decision making by remote monitoring and assessment.

Despite the success of existing approaches, current efforts only focus on the feedback system for the data obtained, especially in the petroleum industry. However, if there is a human system interface, it becomes significantly important to obtain an idea about the human performance and establish a feedback system to caution the operators’ dealing if it is deviating from the standard procedures.

The prospect of real-time data monitoring relies on the performance of the involved personnel. The success of such a system in a complex and highly interactive system like oil and gas relies on the dynamic mental and physical performance of the personal and several related human factors. The behavior is directly implicated by the situation prevailing on the site as well as the personal or social circumstances. The in-situ distractions during real-time data monitoring, especially in the well logs and well operations are eminent at the rig site.

**Figure 1.**
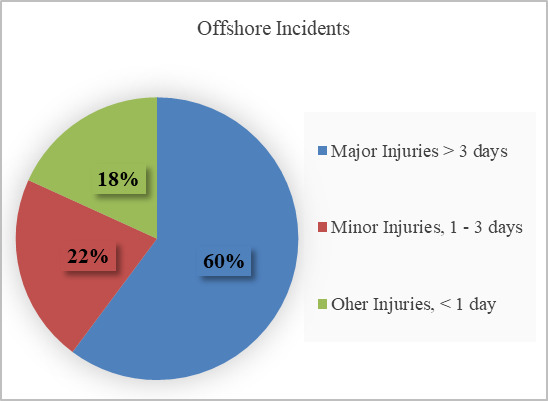
Offshore incidents in the Gulf of Mexico; Major = More than 3 days away from work or more than 3 days of restricted work or job transfer (collectively referred to as DART); Minor = 1-3 DART; and Other = Injuries that resulted in less than one DART (or those that required evacuation to offshore or to another offshore facility for medical treatment but did not result in any DART). (8)

In this context, human errors become the focal point of every accident. Internal data review and analysis by Bureau of Safety and Environmental Enforcement (BSEE) suggest that human engineering problems (e.g., human-machine interface, poor working environments, system complexity, and non-fault-tolerant systems) and problems in work direction (e.g., poor planning, site preparation, selection of workers, and supervision) are the primary drivers of the rate of injuries (8). The injury rates for the calendar year 2018 are depicted in Figure 1 (9). These factors need to be addressed through industry vigilance and strong regulatory oversight to avoid continuous injuries or accidents. With regards to this, feedback systems are highly recommended to supplement human performance.

The underlying human-centered problems during the real-time operation can be eliminated by empowering the system element with capability to alert in case of near-miss environments. The eye tracking system has shown huge promise in several sectors and has become readily available and affordable in the form of portable devices. The oculomotor activity of the participant/onsite personnel can be registered in real-time. This will make the use of this device in the realm of human interactions with the system as well as other fellow professionals in virtual reality setting possible. Analysis of the real-time ocular movement data can hugely complement the human system interface performance. In high-risk environments, the alert system and dynamic feedback system will empower the struggling professionals or trainees to discharge their duty efficiently and accurately.

This paper attempts to bring forth a framework to implement such technology in the oil and gas operation. Before delving into the framework details, it is important to understand the nuances of operations and human factors depicted under process safety and situational awareness.

### Process Safety

Offshore energy extraction operations are complex in nature and consist of several interlinked complex processes. The offshore operations include a complex environment composed of a diverse workforce with different backgrounds. To prevent and minimize accidents, it becomes vital to select and implement a holistic process safety system in the daily drilling operation. Real-time feedback on the crew response to an ongoing activity can help to minimize potential damage. Detecting a deviation of crew attention as early as possible can help the crew to focus on implementing well control procedures before it escalates into a catastrophic event. Review of loss of well control (LOWC) incidents and blowouts while drilling and tripping showed common systemic risks in offshore drilling especially in deep water environments where the late response can escalate to deadly blowouts. Recent ExproSoft report to Board of Safety and Environmental Enforcement (BSEE), summarizing the study of LOWC in the Gulf of Mexico, revealed that the frequency of LOWC incidents is more than that of the workover/intervention operations (27). A robust process safety system can be implemented by integrating both technical and non-technical skills (NTSs). Technical skills are addressed on a regular basis by industry and academicians in depth.

However, the NTSs have only caught the attention of researchers and industry professionals significantly after the catastrophic incident of Deepwater Horizon. The NTSs including communication, situational awareness, risk perception, teamwork, and decision making, play a vital role to avoid the breakdown in the operation process. For example, workers need to communicate with people from different companies as well as their supervisors and management team. Often the professionals make quick decisions especially on LOWC accounts where any loss of attention span increases risks and may cause disasters. No advancement in technological aspects of well control, drilling tools, and machinery can replace a timely expert decision-making process during an event of critical LOWC and blowout. Communication barriers between the operators, contractors and service company’s personnel increase the odds of dangerous and costly decisions. The offshore workforce mix is diverse, and their communication styles differ extensively. Many technical and non-technical issues can bias a person’s situation awareness (55). This can be a challenge to the necessary process safety system which can break down due to lack of understanding.

Human factors play a prominent role in the performance and operational non-productive time (NPT) in drilling operations (53). J. Thorogood and Crichton (54) suggested that important characteristics of high-reliability organizations’ achievements in understanding non-technical aspects from other industries, can be applied to the oil and gas industry. Crew resource management was the type of training adopted from Aerospace industry in late 1970’s by looking at Pilot’s non-technical incidents and the role it plays in airline incidents (43). Recently, crew resource management concepts have frequently been applied in the oil and gas industry. However, the effectiveness has not been established full-fledged. These limitations lead to the problem of quantifying these aspects of NTSs objectively.

Fletcher, McGeorge, Flin, Glavin, and Maran (19) suggested that a comparative study of expert and novices’ behavior can provide a significant impetus to understand non-technical skills. Situational awareness of experts and novices differs in terms of their ability to perceive and interpret the information available in a given situation. Novices, because of their lesser experience and domain knowledge, may exhibit limited ability to detect and interpret relevant cues available in the environment and hence show lower SA. In contrast, the more experience and domain knowledge enables experts to assess situations and make decisions quickly and accurately. Naqvi, Raza, Ybarra, Salehi, and Teodoriu (40) noticed some important differences between the psychological mind states in communication between expert and novice workers. The comparative study relies on the objective measurement of any process safety aspect. Eye tracking technology has a great opportunity in this arena since it can provide quantitative cues on cognitive responses of the participants. These cognitive responses help in understanding the situational awareness of the participants.

### Situational Awareness

Human factors have been attributed to the primary cause of major accidents in high-reliability organizations. Situation or situational awareness (SA) is an important aspect of human factors. Situational awareness is defined as the sum of operator perception and comprehension of associated information, and the ability to effectively project future states of the system using it (30). One of the most popular frameworks for situational awareness is developed by Mica R. Endsley (16) which comprised of three levels: perception, comprehension, and projection. The first level perception includes scanning, gathering the information, and detecting the underlying cues about the prevailing situation in the spatial and temporal domain. The next level, comprehension, consists of the understanding of gathered information and connecting the dots to create mental models. Finally, the third level includes the projection of future circumstances which accompanies the ability to make the final decisions.

The process control system including offshore operation share persuasion of multiple goals by the operators simultaneously, multi-tasking competing for the attention, and performance under severely stressful environment. Failure in the exhibition of these characteristics can be due to difficulties of the acting operators’ SA. Poor situational awareness can slow down the process of problem identification, diagnosis, and reaching a solution which might result in an untimely response (17). Hence, the failure at the level of human performance can be attributed to inadequate situational awareness. In this context, the major challenge is to develop tools that can measure the situational awareness and cognitive state of the participant in real-time.

Several subjective approaches including memory probe techniques and questionnaire analysis had been adopted to measure the responses in the past and some of the important techniques include Situation Awareness Global Assessment Technique (SAGAT) (15) and Situation Awareness Rating Technique (SART) (52). Raza et al. (47) proposed a simulation-based training framework for offshore drilling operations. However, the use of subjective measures is insufficient and will be impossible to connect with RTOC. RTOC can only rely on objective measures. One of the promising technologies regarding objective measurement of situational awareness is eye tracking.

### Eye Tracking

Eye tracking method has been widely used in several industries in the commercial and academic settings to assess the movement of eye activity and patterns of visual search (25). These visual search patterns associated with static and dynamic images have been deemed useful in understanding human behavior, thinking, and decision-making process of the observers. The visual activity is measured using the commercially available eye trackers such as the products sold by Tobii, IMotions, and SR-Research.

The eye trackers use Pupil Center Corneal Reflection technique in which an infrared light source is directed towards the eye and reflections from eyes are recorded by a camera. A specialized camera collects these reflection patterns with added visual information. Then Image processing algorithms are used to generate features such as eyes and corneal reflection patterns. Finally, the gaze point on the screen is calculated using the spatial position of each eyeball (56). There are usually two types of eye tracker available: static monitors, and dynamic eye tracking glasses.

**Figure 2.**
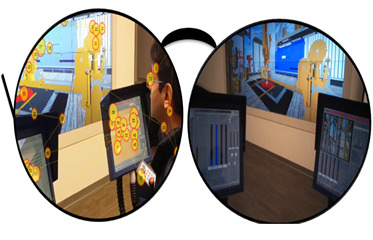
Eye tracking during drilling simulation at the Virtual Reality Drilling Simulator facility at the University of Oklahoma. Visual scan path data overlaid onto an operator’s view of sight: The center of the circles indicate the eye fixation location, the numbers in the circles indicate the time order of the eye fixations, the size of the circles indicate the eye fixation durations, and the linear lines indicate the fast saccades among the eye fixations. Visual search behaviors can be investigated using visual scan paths.

These eye tracking systems provide different types of data including eye fixation duration, fixation count, fixation rate, scan paths, and saccade duration of individuals or group of individuals in a fixed or dynamic area of interest (AOI). An AOI on a static screen-type monitor for eye-tracking is represented in Figure 2, where the center of a circles indicates the eye fixation location and the numbers inside circles indicate the time order of the eye fixations. The size of the circles indicates the eye fixation durations. These circles are connected through straight lines. The straight lines indicate the fast saccades among the eye fixations. The sequence of the eye fixation data constitutes the scan paths which indicate the movement of eye fixations of the participants from one position to the other. These scan paths are useful to investigate the visual search behaviors of the participants. The number of fixations per second is accounted as fixation rate. The saccade duration is a transition time between successive fixations. This data can be further used to establish the cognitive behavioral pattern and identify the objective measures of situation awareness of the participants (17). In addition, metrics-based systems can also be implemented to compare the performance of different participants. Atik, (2) and Atik & Arslan, (1) explored the use and effectiveness of eye tracking technology to assess situational awareness in two different maritime training contexts. In their studies expert participants demonstrated higher situational awareness and more efficient and effective use of electronic navigation aids than novices, as evidenced by their longer fixation durations on relevant AOIs and shorter fixation durations on these aids compared to novices.

Another important aspect of eye tracking data analysis is based on pupillometry, which involves the analysis of the pupil diameter data of both the eyes of the participants. Pupil size is considered as another robust measure of cognitive load and dynamic measure of the processing by the participants while tracking the real-time data. Several studies suggest that pupil size is directly related to stimuli and brain activation distinct with the level of cognitive processing (4). Pupillary dilation has been used to assess the cognitive load (33). Pupil dilation measures have also been used in the medical industry to track the memory load and cognitive response of patients (22). Satterthwaite et al. (50) implemented pupillometry to investigate human decision making. Based on the evidence, we propose that pupillogram may be used to assess participants’ cognitive state during oil well drilling operation, and that may further be used to design a feedback system to enhance process safety in high-risk offshore environments.

### Industrial Applications of Eye Tracking

Eye tracking technology has been used to understand the mental processes in several industries. Study based on flight simulation validated that the dispersion of eye fixation can act as an indicator of mental workload (11). The frequency of more extended fixation is shown to be directly proportional to the workload (59). Research also showed that the participants’ ocular behavior becomes structured in higher difficulty level workload while it stays random in lower difficulty level (14; 51). By analyzing the visual scanning behaviors during an incident, it is possible to identify when and how human factor issues begin to emerge, whether specific visual search behaviors affected their reactions to the incident, and whether crucial data were overlooked.

One of the important avenues in which eye tracking has been widely implemented is air traffic management systems. The analysis of the participants’ ocular activity is used to get insight into the cognitive processes in the flight simulation environment. Several experiments have been conducted involving the scanning of aircraft, identifying and solving the conflicts in the spatial framework (37). The simulated experiments aid the understanding of underlying cognitive processes of controllers and pilots. These understandings led to concepts that can envisage the decision-making strategies between controllers and pilots and pave the path for automated decision aids. Kang and Landry (32) developed an algorithm to interrogate dynamic multiple element objects in the air traffic control display to address the issue of eye fixation mapping in the overlapping AOIs. These techniques can help in automation of eye tracking data processing and develop visualization techniques. Eye tracking has shown to have significant potential in training and selection of pilots (3; 48; 60)

In past decades, eye tracking analysis has also established its application in weather forecasting systems. These studies helped in understanding the underlying cognitive processes of the weather forecasters while detecting the hazards and can be used to improve the situation awareness in the future participants. The reader is encouraged to visit the website for Human Factors & Simulation laboratory at the University of Oklahoma (https://humanfactors.oucreate.com/research.html) for details of various eye-tracking applications in different industries.

Other avenues where eye tracking analyses have been popular are the medical industry. Surgery is a complicated process in the medical profession where effective teamwork becomes the yardsticks for success. The ocular movement analysis of the personnel in the surgery room has been implemented to understand the complex cognitive processes and their complexity during surgery (57; 61). Besides that, eye tracking analyses have also been implemented recently in Human Factors course at the University of Oklahoma, where the real-time scenarios were simulated for well control training (13).

**Figure 3.**
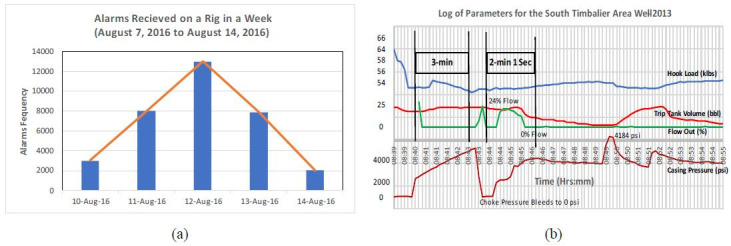
(3a) The alarms received on a drilling rig in a week. The drillers can be inundated with this much information (After Goetz, 23); and (3b) A case study showing the parameters during LOWC and fire in South Timbalier area-2013 (After BSEE, 7)

The use of eye tracking for performance enhancement is increasing across different sectors. Eye tracking has long been utilized in driving and navigation training simulators. (24). Recently, there is evidence of using eye-tracking for expertise assessment and expert-novice differences in visual arts, poetry, and music (20; 35; 46).

### Application of Eye Tracking System from Offshore Rigs to Real Time Operation Centers

Oil and gas drilling process includes highly sophisticated and physically and mentally exhausting operations. Simultaneous running of several procedures and tasks makes it even more challenging. In a typical offshore drilling cabin where the driller continually monitors different gauges and must process huge amounts of information. Most of the routine operations require the driller to be alert of changes in the indicators (such as torque, hook loads), operational parameters (such as weight on bit (WOB), revolutions per minute (RPM)), and more importantly primary kick indicators (such as pit levels and flow). The repetitive and similar set of such data induces boredom in the operators and can act as distracting agent. There is a greater likelihood of an error in such intensive environments. The role of situational awareness becomes crucial for the success of a daily drilling operation (10).

Although alarms are currently employed on offshore rigs to alert drilling crew for potential hazards and they are an essential operator support system for managing abnormal situations, however, they are not very efficient. The status of alarming systems reveals that crews are inundated with several different/types of alarms. Therefore, it is challenging to prioritize them in a way that is taken seriously. The Deepwater Horizon oil spill incident provides an excellent example of an application related to alarm deficiencies. The alarm system guidelines suggest lesser than one alarm per minute as very likely to be unacceptable and one alarm per ten minutes as very likely to be acceptable (42). In a typical drilling operation, as presented in Figure 3(a), the drillers are inundated with around 26,000 alarms over a week (23), and most of them can be ignored. This is highly entangled with the human capability to interact with alarm systems and represents a great human-factors related challenge in control rooms (5). Ikuma, Harvey, Taylor, and Handal (28) suggested a guideline for assessing the performance of petrochemical control room operators. Kodappully, Srinivasan, and Srinivasan (34) used eye fixations to gain insights into the cognitive behavior of control room operators that could be used to proactively tackle human errors.

Figure 3b shows a case study of loss of well control (LOWC) incident from South Timbalier area of Louisiana happened in 2013 (7). A panel investigation concluded that one of the causes of 2013 LOWC and fire in South Timbalier area was: “Failure of the Rig-floor Crew to recognize the loss of well control in a timely manner made it impossible to follow the well control procedures which called for stabbing the safety valve on top of the work string as an initial step.” “The initiation of the emergency procedure sequence to activate blowout preventer (BOP) elements was delayed because of the Rig-floor Crew’s failure to recognize the LOWC at an early stage.” (7). This case clearly reveals that human errors and situation awareness/alertness are crucial in such high-risk operations. Recently, digital data has become an integral part of the petroleum industry. With the advancement of sensors, an enormous amount of quality data is generated and processed within a fraction of a second. In such an environment, eye tracking can be a useful tool to improve the efficiency and accuracy of such an operation. In case the driller is tired and loses the attention span from the monitors, warning alarms can be implemented using real-time eye tracking to alert the driller.

### Application for Real Time Operation Center

Remote real-time monitoring is an interesting and useful technological advancement which is used to look at offshore operations remotely. This is common in the offshore oil and gas industry, particularly for monitoring higher-risk drilling and production operations. This technology is typically used to monitor all aspects of drilling and production operations, including downhole parameters measured while drilling, and readings from the blowout preventer, and the drilling fluid handling system. Shore-based monitoring facilities are often used to analyze data streamed from the higher-risk offshore drilling and production platforms, especially floating platforms in the Deepwater regions of the Outer Continental Shelf. These monitoring facilities are often staffed with highly trained and experienced drilling and production experts, who can view the same data that is being monitored in the Control Room of the offshore platform. The staff at the monitoring facilities provide “a second set of eyes” for observing the offshore operations and can also assist by providing technical and troubleshooting support.

**Figure 4.**
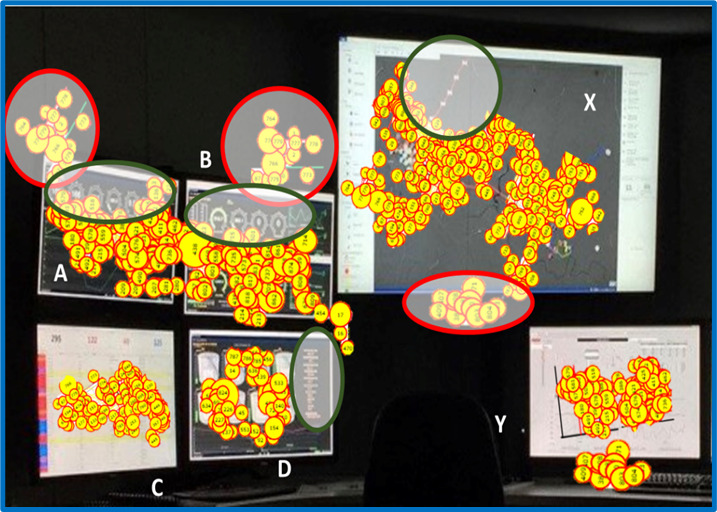
Eye tracking data at real-time operation center (RTOC).

Figure 4 presents a typical offshore RTOC equipped with an eye tracking system. The utilization of multiple screens might distract the operator from effectively performing multiple tasks simultaneously. The eye tracking data depicts the attention span of the operator at the terminal. In Figure 4, the green circular block represents the spatial domain where the operator attentions are missing. The red circular block represents the distraction during the operation. It might be possible that the attention of the operator might have shifted from green circled area to red one. Further, the distribution of ocular movement data is non-uniform. Having such distinct features of this eye fixation data can provide cues which can act as a reliable input for state-of-the-art algorithms to cater with the on-site distractions.

The mental states of an alert and tired person are entirely different. A tired person could feel sleepy and may close his eyes. The basic premise of the eye-tracking technology relies on recording of light reflected from the cornea. The sleepy person will frequently close its eyes, and the eye tracking camera will not be able to capture the fixation data or pupil size. This very concept can be utilized in the realization of the difference between the scanning behavior of alert and non-alert person. The eye fixation count and duration will be significantly missed during such conditions. The comparison of eye-fixation count and duration of the subject and standardized expert data over a particular real-time monitoring operation can provide a cue towards the state of the operators. The decrease in pupil size in a real-time system can also provide cues on alertness of the subjects. If the ocular activity of the onsite participant is below a satisfactory level, the system can be triggered. The triggered event can alert the participant to be cautious about the AOI which lacked the focus of the participant. Keeping this in mind, establishing the working procedure of eye tracking technology with regards to oil and gas operation with different mental state of the participants becomes highly desirable. Hence in the next section, an experimental investigation with data analysis is presented to extract the difference in situational awareness quotient of different participants.

## Methods

This study used a controlled simulated environment consisting of eye trackers, display screen, and well-log data to measure SA. The participants that we define as novice and an expert were presented a scenario of a real-time well-log data of an oil well on the display screen, and were tasked to monitor the real-time data, interpret the data, explain their understanding, and point out any abnormalities while their eye movements were being recorded using an eye-tracker. The experiment was conducted using the Virtual Reality Drilling Simulator (VRDS) available at the University of Oklahoma.

Some AOIs were defined on the well-log data. Then, the eye fixation durations and counts were collected during the simulation for all the participants. The data were processed to obtain the percentage of eye fixation durations and counts for further analysis. “Percent eye fixation duration” represents the percentage of the eye fixation span of participants at an AOI over the total fixation duration considering the whole log as an AOI. Similarly, “percent eye fixation count” refers to the percentage of fixation counts in an AOI with respect to total counts in the whole log. The percentages of fixation duration and fixation count were used to normalize the difference in the plot area of the considered AOI. Then a statistical study was conducted to obtain cues on the cognitive response of the participants.

The participants were given a basic knowledge of drilling scenario and human factors. The novices were trained for a theoretical understanding of influx indicators and power loss on the rig. Before simulation, a pre-briefing was conducted where the details of the scenario, execution procedure, and the expected responses were explained. The scenario was limited to five minutes. During the simulation, the participants’ gaze movements were captured using eye trackers in real-time on the pre-defined AOIs. We hypothesized to observe differences in the ocular activity of the expert and the novice.

### Participants

For this research, we recruited twenty-three undergraduate and graduate students of the Petroleum engineering department at the University of Oklahoma as novices. These participants were given classroom training about the drilling operations and well control procedures prior to the experimentation. In addition, we had an expert participant from the industry who had more than 30 years of field experience of working on oil and gas rigs. In total, this study involved twenty-four participants.

**Figure 5.**
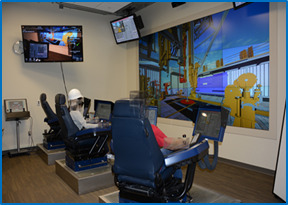
Virtual Reality Drilling Simulator (VRDS) at the University of Oklahoma.

In this paper, we compared the ocular activity of a novice (one of the twenty-three novices) and an expert as indicators of situational awareness to show the potential of using these differences for a feedback system at an RTOC.

### Materials

**Figure 6.**
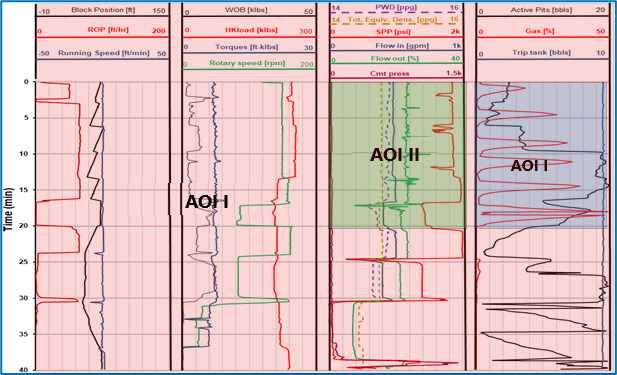
Area of Interests on the dynamically displayed well logging data (after NorskOlje&Gass (41)).

The study was conducted using the VRDS facility at the University of Oklahoma. The VRDS room is equipped with the National Oilwell Varco (NOV) offshore drilling simulator, and it closely mimics the offshore operational environment. This multimillion-dollar facility simulates an offshore environment of a Drillship with three cyber base chairs as shown in Figure 5. The cyber chairs have two display monitors (one on each arm), keypads, roller-mouse, and joysticks to control the equipment and perform common surface operations on a rig such as Tripping-in, Tripping-out, Top-drive to Pipe connection, and Drilling. The facility also has joystick module to operate pipe and riser catwalk. The display wall is used for visualization of an oil rig platform. For this simulation and eye tracking, a Visual Basic program was written to display the data in real-time on the monitor screen which included the different parameters as exhibited in the scenario plot shown in Figure 6. All the data was displayed in the form of lines with different colors, and a legend was provided to depict the characteristics of the lines.

The eye tracking was captured using Tobii TX 300 eye tracker with a sampling rate of 300 Hz and a visual angle accuracy of 0.4 degrees. The eye tracker was placed approximately three feet from the participants’ eyes. A 23 inches monitor was fitted above the eye tracker to display the log of the scenario. The data was recorded and digitized using Tobii Studio 4 software and then later analyzed using R software.

### Procedure

Prior to this simulation study, the novices were given training on reading and interpretation of well logs and to identify the abnormalities. For this scenario, the protocols for pre-briefing, simulation, and debriefing were developed. The pre-briefing included training on different aspects of well log parameters and simulation practice. After that, the participants were briefed about the procedures and standard practices of the scenario simulation.

The participants’ ocular activity was monitored using Tobii eye tracking monitor, and the data was collected. The participants were instructed to interpret the displayed dynamic well data. The primary task was to identify the abnormality in parameters, in-situ conditions, and forecast the consequences. The objective of this scenario was to assess the participant’s cognitive behavior during the real-time data monitoring of well-logs.

### Data Collection

Eye movement data for eye fixation duration, eye fixation count, and pupil size during the scenario were collected using Tobii eye trackers in a single trial. We defined three AOIs to capture eye movements data as are shown in Figure 6. The full log is considered as the AOI I less the other two AOIs. Apart from this, two spatially fixed AOIs were designed to correspond to the real-time monitoring of participants’ response. These two AOIs are represented as AOI II and AOI III. The AOIs were selected based on their support as indicators for the interpretation. The major indicators include rate of penetration (ROP), flow out (Flow out %), gas penetration (Gas %), and active pit volume (Active Pits). The ROP, which represents the speed of penetration of the drill bit into the rock to deepen it, is the most important indicator as it would increase in possible situations of a kick (a rapid increase in oil well pressure or gas influx). However, this alone cannot be used to confirm the kick as it could be due to softer strata other than gas influx. Therefore, the operator needs to confirm it through Flow out %, Gas %, and Active Pits. AOI II covers sudden picks in the Flow out %. AOI III covers Gas % and Active Pits.

The eye movement data was registered and stored in Tobii Studio software which included the spatial fixation and 0.42 pixels/milliseconds velocity threshold (32). The analysis of these AOIs was conducted using simulation output and eye tracking output.

## Results and Discussions

**Figure 7.**
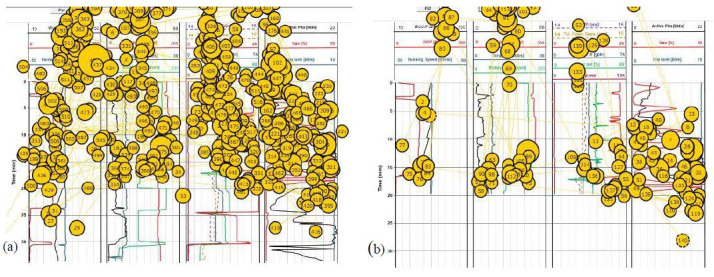
Ocular data distribution of the expert during the full duration of the simulation 7(a) and during 2:00 to 2:50 second interval 7(b).

**Figure 8.**
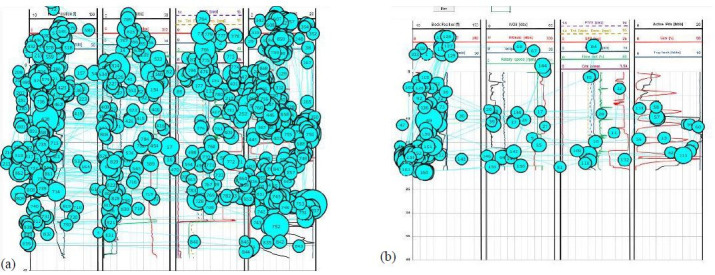
Ocular data distribution of the novice during the full duration of the simulation 8(a) and during 2:00 to 2:50 second interval 8(b).

The sample eye fixation data for the expert is shown in Figure 7, whereas the data of the novice is shown in Figure 8. The data consists of various sizes of circles with different numbers inside it. The size of these circles represents the amount of time span of the participant at a particular spatial position during the simulation. The visual search/scan behavior differs from participant to participant. To account for that, the eye tracking software implements the timestamping of each fixation. Corresponding to the time stamp, the software assigns the numeric order. The number inside the circle represents this sequential numbering of visual search. In this study, only the fixation spans and counts are accounted to conduct the assessment of participants’ attention while scanning the plot in real-time.

The eye fixation data in Figure 7(b) and 8(b) was collected for a 50-second duration, during a critical time period from 2:00 to 2:50 seconds, where an anomaly (a kick) was present, and the participants were required to properly identify it and take appropriate actions to verify and control it.

The preliminary investigation of the overall distribution of scanning behaviors among participants shows a significant difference from one sector of the plot to the other. The data shows that the expert correctly detected the anomaly and is providing proper inputs to mitigate the anomaly, whereas the novice continues observing the graph as the eye fixation data is all over the graph. During the critical 50 second duration, the expert correctly creates eye fixations on the anomaly that required attention (i.e., AOI III), whereas the novice does not with minimal eye fixation in the AOI III.

Further, it is seen that during the critical 50-second interval, the expert is well aware of the situation, as evidenced by the eye tracking data (i.e., eye fixation in AOI III), whereas the novice seems to be too much occupied on the left end of the graph (continuously observing ROP) and not confirming the anomaly through other indicators in AOI II and AOI III which can be inferred as low situation awareness of the novice. From the full-duration eye fixation data, it seems that the expert knows it is meaningless to continuously observe further (which accords with why we chose such AOIs for analyses) and concentration the anomaly detected in AOI III, whereas the novice does not really know what is going on.

It is worth noting that a real-time alarm/feedback system, which can caution the participant about this deficiency, will improve the performance of the subject. We hypothesized that the situation awareness will be different between the novice and the expert based on the assumption that the situation awareness is correlated with eye fixation data which is well supported in the literature. In detail, the hypothesis assumes that the participant with greater situational awareness will have greater fixation data on certain AOIs. This hypothesis is also substantiated by the high correlation and similar trends observed in the two categories with respect to eye fixation duration and count data.

There is a considerable amount of scientific evidence to support the hypothesis that situation awareness is correlated with eye fixation data and that expert participants may exhibit greater situational awareness than novice participants. has been supported by several studies in literature. Studies have shown that eye tracking data can be used to assess situational awareness and training effectiveness in various industries, including aviation, healthcare, military settings, and oil and gas operations. For example, a study by Endsley and Garland (18) found that expert pilots had greater situational awareness than novice pilots, and that this was reflected in differences in their eye gaze patterns. Similarly, Salehi et al. (49) developed a cross-disciplinary, scenario-based training approach integrated with eye tracking data collection to enhance situational awareness in offshore oil and gas operations. The authors found that eye tracking data could be used to identify areas where situational awareness was lacking and to tailor training interventions accordingly. Similarly, Kang, Jeon, and Salehi (31) used eye tracking analytics to evaluate the effectiveness of virtual reality training for situational awareness. The authors found that eye tracking data could be used to measure trainees' visual attention and identify areas for improvement in the training program. Hermens, Flin, and Ahmed (26) provide additional evidence to support the idea that there are differences in situational awareness between novices and experts and that experts tend to have more focused and efficient eye movements compared to novices.

**Figure 9.**
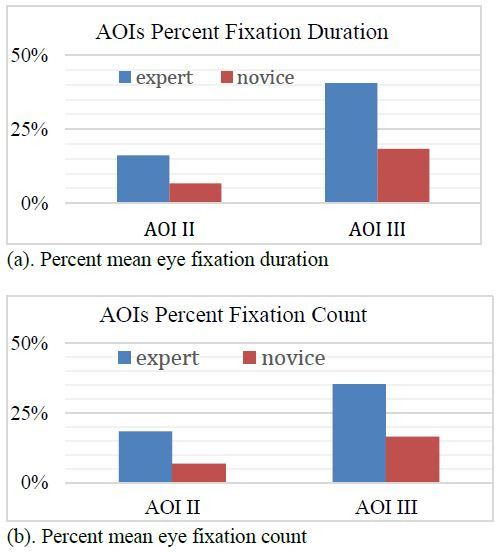
Percent mean eye fixation duration 9(a) and count for the novice and the expert 9(b).

The oculomotor data for the mean percent eye fixation duration and count for the novice and the expert are provided in Figure 9. The comparative study of participants’ response between two AOIs suggested that participants tended to focus more on AOI III than the other AOIs. Figure 9(a) depicts the visual representation of participants’ data for mean percent eye fixation duration. The significant difference in response between AOI II and AOI III can be explained by the deviation of the log parameters in both AOIs. The gas percent fluctuation is more prominent in AOI III than other parameters of the log which might have caught the attention of the participants. This pattern suggests that the participants’ visual attention can be correlated with their situation awareness.

Similar observations were made for the percent mean eye fixation count data as shown in Figure 9(b). The novice was found to be on the lower side compared to the expert fixation counts in case of AOI II and AOI III. This trend can be attributed to distraction exhibited by the less situationally aware participant. The significant differences of scanning behaviors between novice and expert on the important AOI III is significantly lower than that of the novice’s duration indicating that the expert was able to quickly assess the situation than the novice.

Apart from visually observing the data, a statistical study was conducted on the visual data where we compared the mean of all novices’ data with the expert to understand the underlying. ­To find out the significance of the difference in fixation duration and fixation counts across three AOIs, non-parametric Kruskal-Wallis test was performed. The results indicated significant difference across three AOIs in terms of fixation duration (H (2, 66) = 51.24, p < .01) and fixation counts (H (2, 66) = 55.39, p < .01). Further, pairwise comparison indicated significant difference in fixation duration of AOI I with AOI II and III, and of AOI II with AOI III (see Table 1a). The same was observed for fixation counts, i.e., significant difference in fixation counts of AOI I with AOI II and III and of AOI II with AOI III (see Table 1b), indicating that there is significant difference in terms of fixation duration and fixation counts on all three AOIs.

While comparing novices’ data it was observed that novices spent more time and had more counts on AOI I which is of lesser importance for anomaly detection and confirmation (see Figure 10a & b). In comparison, the expert had much lesser counts in this AOI, clearly showing that he knew where to look in case of an anomaly detection.

**Table 1a.** Pairwise Comparisons of three AOIs on fixation duration

Sample 1 - Sample 2	Test Statistic	Std. Error	Std. Test Statistic	Sig.	Adj. Sig.^a^
AOI II - AOI III	-22.727	5.788	-3.927	.000	.000
AOI II - AOI I	-41.364	5.788	-7.147	.000	.000
AOI III - AOI I	-18.636	5.788	-3.220	.001	.004

**Table 1b.** Pairwise Comparisons of three AOIs on fixation counts

Sample 1 - Sample 2	Test Statistic	Std. Error	Std. Test Statistic	Sig.	Adj. Sig.^a^
AOI II-AOI III	-20.091	5.788	-3.471	.001	.002
AOI II-AOI I	-43.045	5.788	-7.437	.000	.000
AOI III-AOI I	-22.955	5.788	-3.966	.000	.000

**Figure 10a.**
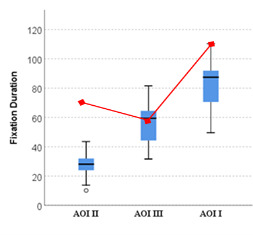
Mean fixation duration on all three AOIs by Novices, with expert’s values imposed in red.

**Figure 10b.**
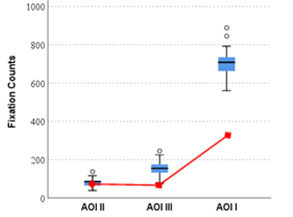
Mean fixation counts on all three AOIs by Novices, with expert’s values imposed in red.

For the AOI II and III, the overall time frame comparisons between novices and the expert did not clearly show significance. This could be due to multiple reasons such as the expert might have immediately noticed the anomaly, acted, then ended (no need to see more), whereas a portion of the novices immediately noticed but took more time by looking it longer, and another portion of the novices did not notice, so averaging them would provide unexpected results. This needs further experimentation and is planned as future research.

Further, the novices might have different levels of situation awareness meaning that some were able to quickly notice the issue whereas some were not. The differences in situational awareness of participants can be due to several factors such as domain specific knowledge, prior experiences, mood, alertness, fatigue, distractions etc. The representative example shows a case when a novice clearly lacked awareness (see Figure 7). The analysis of each participant's awareness level has not been analyzed in this paper, and an in-depth and systematic method is being devised to better classify various awareness levels as future research to corroborate.

### Pupillogram Analysis

Apart from the fixation data, pupil dilation of the participants was also studied. The main steps involved in this data analysis are noise filtering from the pupil size data, averaging both (right and left) pupil size values, baseline adjustment, and then normalizing the data. First, one of the signal smoothing and noise filtering techniques, Savitzky-Golay filter was implemented to minimize the error for excessive sampling at the onset or offset of gaps. Savitzky-Golay filter is one of commonly used low pass filtering technique in signal processing (12; 36). In addition to this filtering technique used for removing the noise from the data, linear interpolation was applied to calculate the missing values of the pupil size. For interpolation, the slope was calculated using a window of 21 data points (10 data points above the missing data and 10 below the missing data) (29). Furthermore, the average value of pupil size of both eyes was calculated. To compare the responses between the participants, the data was normalized after baseline adjustment.

**Figure 11.**
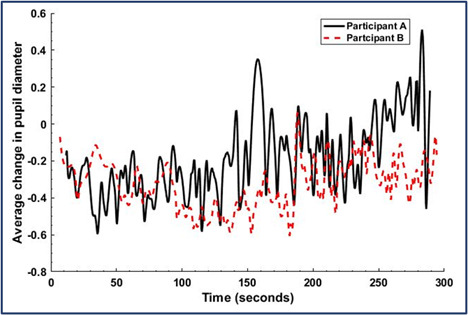
Average change in pupil diameter (millimeter) of novice and expert while monitoring the real-time data during scenario execution.

Figure 11 shows the dynamic pupil size distribution for the novice and expert. On a closer look at the plot, it is evident that both participants have similar change of pupil size in the beginning. However, the novice exhibited less dilation in pupil size during 100-200 seconds of the experiment. Again, in the range of 200-250 seconds both participants’ responses were almost similar. However, in the final 50 seconds the dilation of the novice was lower than that of the expert. As explained in the previous section, pupil dilation measure shows better cognitive response from the expert which can be inferred as better alertness and awareness. In addition, when the data of AOI II and III were combined for both participants, the percentage of fixation exhibited similar characteristics suggesting a high correlation between the eye fixation data and the pupillogram.

Overall, we can infer that the explicit distinctions between the eye movements of experts and novices can be used to set up a framework that can characterize the alertness and awareness of the participants. The eye tracking data can also be used to provide feedback while the users are tracking the data in the RTOC. For example, if the eye movement characteristics deviate from those of a representative expert, then an alert can be issued, or scaffolding materials can be provided in a timely manner.

## Conclusion

A study of the real-time monitoring system with regards to the oil and gas industry has been conducted. In addition, the use of eye tracking technology in various applications, such as aviation, weather forecasting, and medical industry, were investigated that can be adapted to offshore oil drilling operations. Based on the theoretical concepts, the role of eye tracking in the real-time monitoring system was explained and resulted in establishing a framework for applying the oculomotor data to better assess operators’ cognitive states. Finally, an experiment using the scenario-based offshore operation simulation was conducted using eye tracking technology.

The simulated experiments exhibited the non-uniform distribution of eye fixation data over the dynamically displayed well-logging data which can be correlated with the situational awareness of the participants. In addition, eye fixation data can be used to quantify the scanning behavior. The study showed promising results which can be further linked to individual situational awareness and be processed in real-time to establish a feedback/alert system. In addition, a pupillometry analysis was conducted and the pupil dilation effect clearly distinguished the cognitive responses of the participants and showed high correlation with the other eye tracking data such as fixation counts and durations.

For this study, we were fortunate to have an experienced field expert with 30 years of experience. It is planned to recruit more experts for future studies. Further, the ranking of participants in terms of performance may be possible with more data. This is planned studies as well. Furthermore, upcoming studies aim to modify advanced techniques for analyzing eye movements, which encompass visual entropy (39), a multimodal approach to analyzing fatigue and eye movements (38), a framework for analyzing eye movements in real-time (45), and machine learning (44). These techniques can be utilized to formulate real-time safety assessment algorithms for various drilling operations at drilling rigs and Real Time Operation Centers.

### Ethics and Conflict of Interest

The author(s) declare(s) that the contents of the article are in agreement with the ethics described in http://biblio.unibe.ch/portale/elibrary/BOP/jemr/ethics.html and that there is no conflict of interest regarding the publication of this paper.

### Acknowledgements

This material was based upon work supported by the National Academy of Sciences, Engineering, and Medicine under the Gulf Research Program (Grant #2000007356). The opinions, findings, and conclusions or recommendations expressed in this material are those of the author(s) and do not necessarily reflect the views of the National Academies of Sciences.

The authors would like to thank Mr. Stan Christman who helped in the review of scenario experiments.

### Abbreviations and Acronyms

*AOI   Area of Interest*

*BOP   Blowout Preventer*

*BSEE   Bureau of Safety and Environmental Enforcement*

*CRM   Crew Resource Management*

*LOWC   Loss of Well Control*

*NOV   National Oilwell Varco*

*NPT   Non-productive Time*

*NTSs   Non-technical Skills*

*ROP   Rate of Penetration*

*RTOC   Real Time Operations Center*

*SA   Situation/Situational Awareness*

*SPP   Standpipe Pressure*

*VRDS   Virtual Reality Drilling Simulator*

*WOB   Weight on Bit*
